# Populist Anti-immigrant Sentiments Taken Seriously: A Realistic Approach

**DOI:** 10.1007/s11158-021-09516-1

**Published:** 2021-05-26

**Authors:** Laura Santi Amantini

**Affiliations:** Department of Classics, Philosophy and History (DAFIST), University of Genoa (Università Degli Studi Di Genova), Via Balbi 30, 16126 Genoa, Italy

**Keywords:** Anti-immigrant sentiments, Right-wing populism, Ethics of migration, Realism, Liberalism

## Abstract

This essay argues that the illiberal anti-immigrant sentiments which lie behind the success of populist right-wing parties deserve the attention of political theorists working on the ethics of migration, even though such sentiments exceed the boundaries of admissible disagreement on justice in migration. Firstly, populist anti-immigrant sentiments hinder the implementation of liberal democratic immigration policies and thus they represent a feasibility constraint for any liberal ethics of migration, not only the most cosmopolitan ones. Secondly, there are legitimacy reasons why such views should be neither merely dismissed nor simply contained, since they are voiced by populist political parties which are admitted in the electoral competition and even participate in governments. The main upshot of this discussion is a methodological one: the article argues that, since political theory should deal with the feasibility and legitimacy issues raised by populist anti-immigrant sentiments, a realistic approach is needed. The last section shows that such a methodological change offers the opportunity to extend the scope of normative theorising. In particular, it illustrates how a realistic approach encourages theorists to focus on local-level policies, as well as to devote attention to non-governmental actors and to their role in tackling citizens’ hostility towards immigrants.

## Introduction

Right-wing populist movements have been gaining prominence in liberal democracies and have become real competitors to mainstream political parties. Normative political theorists have started to consider the challenges that populism (especially right-wing populism) poses to liberal democracy and how to address them (see Wolkenstein 2015; Badano and Nuti 2017; Ferrara 2018; Mouffe 2018). However, the illiberal anti-immigrant sentiments that lie behind the success of contemporary right-wing populist parties in Western liberal democracies have not yet gained much attention in political theory.

This paper will approach anti-immigrant sentiments from the angle of the liberal ethics of migration. I will argue that populist anti-immigrant sentiments pose a formidable challenge to justice in migration and should not be dismissed. Indeed, they provide popular support for immigration policies and practices incompatible with the basic values of freedom and equality. Nevertheless, they cannot be simply contained and condemned either, given that they are voiced by political leaders and parties that regularly participate in elections and are allowed to govern. Thus, political theorists should seriously assess how populist anti-immigrant sentiments should be tackled. Although normative theorists should be realistic in acknowledging such sentiments, they should not assume them to be an immutable feature of the real world. Instead, I will argue that they should adopt an empirically informed approach to assess how these sentiments arise and how they can be effectively and legitimately addressed by institutions and civil society. This, I will show, opens up promising new avenues for the ethics of migration by highlighting the key role of local-level policies and practices.

The first section will argue that populist anti-immigrant sentiments are morally problematic because they exceed the bounds of what is admissible as mere disagreement among views of justice in migration grounded on the basic liberal values of freedom and equality. Thus, they challenge not only cosmopolitan theories of justice in migration that defend open borders but also liberal theories that, despite defending a qualified right to exclude immigrants, are committed to basic liberal values. ‘Right-Wing Populist Movements and Anti-immigrant Rhetoric’ section will show that there are important reasons why political theorists should deal with populist anti-immigrant sentiments as such, despite such sentiments being illiberal. First, they hinder the implementation of liberal democratic immigration policies, and thus they represent a feasibility constraint for any liberal ethics of migration—not only for the most cosmopolitan ethics. Second, addressing these sentiments induces legitimacy constraints.

The main point of the paper is methodological: ‘Populist Anti-immigrant Sentiments in the Ethics of Migration’ section will argue that a realistic approach to the ethics of migration is needed to acknowledge the widespread illiberal anti-immigrant sentiments promoted by populist parties and to recognise that they raise normatively relevant feasibility issues and democratic legitimacy issues. Realistic normative theorising will be defined as a methodological, rather than substantive, perspective, which need not assume that real-world constraints are equally binding and permanent. The section will not attempt to offer a full-fledged realistic theory of justice in migration, which would certainly need to include several real-world constraints in addition to anti-immigrant sentiments. Instead, it discusses why and how a theory meant to be realistic should deal with the reality of anti-immigrant sentiments. Being realistic about anti-immigrant sentiments means acknowledging their influence and the moral issues they raise but also looking at how they are formed and the extent to which they are malleable. This is highly relevant for assessing the role that such real-world facts should be given in theorising just and permissible immigration policies. Furthermore, a realistic approach should consider which actors can influence citizens’ sentiments about immigrants and what they should do in the face of the widespread illiberal sentiments supported by populist parties.

Indeed, ‘Dealing with Anti-immigrant Sentiments in Normative Theorising: a Realistic Approach’ section will illustrate how a realistic approach allows normative theorists to recognise the crucial role played by local-level governments and non-governmental actors in addressing citizens’ attitudes towards migrants. This is an important theoretical development since it broadens the scope of the ethics of migration policies beyond nation-level admission and integration policies.

## Right-Wing Populist Movements and Anti-immigrant Rhetoric

Right-wing populism has become very influential in liberal democracies, gaining electoral success in Western and Central Europe^1^ and proving to be even stronger in Eastern Europe’s younger democracies.^2^ While in the US, Donald Trump’s election in 2016 was seen as an unprecedented phenomenon, right-wing populist parties had already obtained remarkable results all over Europe in earlier elections, and their appeal does not seem to have been substantially undermined when they ceased to be outsiders and began to participate in government. Thus, their success is neither episodic nor a mere expression of protest. Indeed, ‘the key to the electoral persistence of populist radical right parties is their ability to transform protest voters into support voters’ (Mudde 2007, p. 229).

Populist right-wing parties appeal to anti-elitist and nativist ideologies, both expressing a form of antagonism between the people and their enemies (Mudde 2007, p. 22; see also De Cleen 2017). The first antagonism contrasts virtuous people with the corrupt elite.^3^ Populist anti-elitist claims aim to replace the values of the dominant Establishment with the common sense of the people (Guibernau 2010, p. 10; Betz 1994, p. 4), as explicitly stated in the League Party’s slogan for the 2018 Italian parliamentary elections: ‘The revolution of common sense’. While anti-elitism is usually identified as a common feature of all populist movements,^4^ another core trait that characterises right-wing movements is antagonism between a homogeneous native people and aliens.^5^ These two antagonisms often overlap: the Establishment is believed to be plotting against the people and siding with threatening outsiders (namely, immigrants) rather than with its own citizens (Bergmann 2020, p. 50; Mudde 2007, p. 66). Authoritarian tones are also utilised to stress a firm rejection of the Establishment’s tolerant and multicultural policies and to call for the restoration of law and order. Right-wing populism is thus marked by a strong anti-immigrant rhetoric, which has emerged as its most noticeable feature. Ironically, such a nationalist and exclusionary ideology has a transnational character, appearing in a similar manner in several countries (Guibernau 2010, p. 13).

There is a mutually reinforcing link between the anti-immigrant sentiments expressed by populist right-wing parties and the anti-immigrant sentiments that spread among citizens. To gain electoral success, populist right-wing parties exploit hostile opinions and emotive reactions about immigrants, which nonetheless do not emerge in isolation: they are shaped, amplified and rationalised by politicians and echoed and strengthened by the media. Indeed, populist right-wing rhetoric and the narratives expressed in partisan media and social networks are responsive to pre-existing sentiments, but they do not merely voice them; rather, they simultaneously ‘reflect and forge’ such sentiments (Polletta and Callahan 2017). Populist right-wing leaders and media crucially play a role in increasing or decreasing the salience of particular issues and creating connections between particular episodes, pre-existing beliefs and emotions (e.g., by capitalising on cognitive biases, by adopting fallacious arguments and implicatures). Furthermore, they rationalise and normalise opinions, behaviours and emotive reactions, including those that are radical or minoritarian, making them mainstream and acceptable to a larger audience (Krzyżanowski 2020). Thus, anti-immigrant sentiments, including blatant racism, are increasingly perceived as morally permissible, even common sensical, paving the way for unjust immigration policies and providing social legitimacy to unjust social practices.

It is true that disagreement on just and permissible immigration policies can also be traced in the academic debate on the ethics of migration. Liberal authors hold diverse positions, from cosmopolitan to nationalist ones, and they disagree on what justice in migration requires in principle, as well as on how states should act under the current non-ideal conditions. The key issue has long been what admission policies should be like. Liberal egalitarian thinkers such as Joseph Carens (1987, 2013) argue that, in principle, justice in migration requires open borders. By contrast, liberal nationalists such as David Miller (2007, 2016a) defend the right of the receiving state to exclude immigrants to protect citizens’ national identity and interests. Integration and citizenship policies are also disputed: theorists are far from a unanimous agreement regarding what is due to immigrants who have already settled. In brief, liberal political theorists disagree on how to balance cosmopolitan impartialist moral claims, which are consistent with the principle of individuals’ equal moral worth, with the partialist moral claims of citizens, which are grounded on the state’s special responsibility towards them (Gibney 2004).

It might then be suggested that right-wing populism is ultimately trying to convey claims for partiality analogous to the claims advanced by liberal nationalist theorists. However, the peculiarity of populist right-wing movements lies precisely in their ambivalence. Such parties generally seem to accept basic liberal constitutional values and are admitted into electoral competitions. Their programmes may also avoid openly racist, dehumanising or inferiorising claims—or may at least nuance them. Nevertheless, they appeal to illiberal sentiments, which emerge in their rhetoric. Although a partiality towards compatriots may be compatible with liberal principles, as Miller contends, right-wing populist propaganda often radically exceeds the limits of what constitutes mere disagreement among interlocutors who accept the basic values of freedom and equality.^6^

Both the content of populist right-wing rhetoric and also the language chosen to express it are aimed at challenging basic liberal principles. Right-wing populist politicians and their supporters may deny any obligation whatsoever towards non-citizens. They may justify the mass refoulement of undocumented migrants before they reach national borders, irrespective of the migrants’ condition or potential refugee status, and the deportation of those who have managed to cross borders, including long-term residents.^7^ Significantly, they label undocumented migrants ‘illegals’, i.e., lawbreakers, and explicitly associate them with criminality and insecurity. They may go as far as dehumanising immigrants by comparing them with animals^8^ and associating them with disgust-eliciting images of unfettered sexual impulses, dirt and disease.^9^ Right-wing populists may also invoke discriminatory admission bans for stigmatised categories of immigrants: indeed, rather than expressing generalised xenophobia, populist anti-immigrant sentiments are usually directed towards specific types of what they see as undesirable individuals.^10^ Therefore, right-wing populists may even deny fundamental rights to national or non-national residents, who are inside the territory of the state but are not recognised as part of the nation.^11^ In fact, members of groups perceived as ‘other’ (e.g., Roma and Sinti minorities, immigrants and persons of immigrant descent, especially Muslims) are ‘depicted as unable to be and even become fully functioning members of society’ (Badano and Nuti 2017, p. 6).

It is worth noting that some supporters of right-wing populist movements may still be unaware that they hold illiberal beliefs and even think that they are actually the guardians of the liberal Western heritage threatened by barbarous invaders (Badano and Nuti 2017), while others proudly declare themselves illiberal. However, in both cases, they typically deny epistemic authority on alternative liberal views on immigration. Anti-elitist sentiments make them suspicious of the moderate tones of academics and mistrusting of the mainstream press, which is thought to be as corrupt as the political elite and to be hiding or minimising the dangers that immigrants’ presence entails (Mudde 2007, p. 67). Therefore, such supporters may rely on sensationalistic tabloids or on partisan niche media, which offer them ‘an experience of being in a conversation’ with someone who accepts their views (Polletta and Callahan 2017). These media thus contribute to reinforcing stereotypes and fostering hostile attitudes.

In sum, populist anti-immigrant sentiments are at odds with the basic liberal values of freedom and equality. Therefore, such sentiments are fundamentally illiberal, despite the ambivalence of the right-wing populist parties that both foster and exploit such sentiments while usually endorsing the liberal democratic state. Thus, the spread of populist anti-immigrant sentiments is troublesome for any theory of justice in migration grounded on liberal values, including those theories that give considerable weight to the partialist moral claims of citizens.

## Populist Anti-immigrant Sentiments in the Ethics of Migration

The diffusion of populist anti-immigrant sentiments has not yet gained much attention in the literature on the ethics of migration. For instance, the issue is not discussed by Carens in *The Ethics of Immigration*, while in S*trangers in Our Midst* Miller only briefly mentions the existence of ‘resentful working-class whites, susceptible to incitement by far-right parties’.^12^ In a recent book, Javier Hidalgo (2019a, b) devotes some sections to how anti-immigrant attitudes are formed and how they can be counteracted, but the book deals with an individual ethics of migration rather than with the role of institutions.

There seem to be two good reasons to think that the debate on the ethics of migration need not consider populist anti-immigrant sentiments as such. One reason may lie in the conception that normative political theory is primarily concerned with what is morally ideal. Some may thus maintain that the ethics of migration are not interested in what ordinary people think about migration but in how states should act according to moral principles. As Matthew Gibney pointed out, normative theorists are inclined to abstract from practical constraints, including political constraints (Gibney 2004, pp. 15–16).^13^

A second reason to exclude illiberal anti-immigrant sentiments from an ethics of migration may depend on their being dismissed as embarrassing and unreasonable. Such claims can be overtly racist and hardly defensible on the grounds of liberal principles. A liberal nationalist thinker, such as Miller, may contend that ‘the general justification for immigration restrictions involves an appeal to national self-determination and in particular a people’s right to shape its own cultural development’ (Miller 2007, p. 228), but he would nonetheless deny that such a principle can legitimise the rejection of a migrant’s claim to entry on the basis of their nationality, ethnicity or religion (Miller 2007, p. 229). In terms of integration policies, Miller maintains that Muslims cannot be refused a mosque to pray in, though he defines minarets ‘unessential features’ (Miller 2016b). At most, right-wing populist sentiments seem to offer a starting point for a more general and reasonable reflection once deprived of their ‘lurid language’ (Miller 2016b).

Nevertheless, in what follows I argue that populist anti-immigrant sentiments should be taken seriously. It has already been noted that the greater the number of illiberal persons, the higher the risk to the stability of liberal institutions (Badano and Nuti 2017, p. 3). On such grounds, Badano and Nuti have argued for a ‘duty to pressure’ falling upon ordinary people—namely, a moral duty to discursively engage with the right-wing populist views expressed by their fellow citizens (Badano and Nuti 2017, p. 12). However, I will not insist on the issue of stability. My point here is that the diffusion of populist anti-immigrant sentiments in particular entails at least two additional implications, which are specifically relevant for liberal political theorists interested in the ethics of migration.

First, the rise of populist anti-immigrant parties results in strong feasibility constraints for a liberal ethics of migration. As I will discuss in more detail in the following section, normative theorising may include different degrees of idealisation depending on the goal of the inquiry. When theorists discuss what justice in migration requires in principle, they may bracket the contingent real-world features that would make their normative conclusions unlikely to be immediately translated into policy. By contrast, when considering how such principles of justice can guide policy and practice, an ethics of migration is supposed to include at least some real-world features that policymakers cannot ignore. The hostile attitude towards immigrants evident in public opinion should be counted among these features. Indeed, such sentiments constrain the implementation of any liberal admission or citizenship policy, however inclusive, since they express strongly discriminatory and illiberal views. Even a liberal nationalist position such as that of Miller would concede too much to cosmopolitan and humanitarian obligations from the perspective of those citizens who endorse populist anti-immigrant sentiments.^14^ It could thus be counterproductive for theorists to dismiss illiberal anti-immigrant sentiments and to urge policymakers to follow the normative prescriptions of a liberal ethics of migration despite a large share of citizens holding such views, since forcibly imposed policies can provoke backlash (Carens 1996, p. 160; Gibney 2004, pp. 212–213).

Second, anti-immigrant sentiments in Western liberal democracies raise important theoretical problems from a democratic point of view. Since they pose feasibility constraints on any immigration and integration policy consistent with the basic liberal values of freedom and equality, there are good reasons to address such sentiments. However, addressing populist anti-immigrant sentiments raises special normative concerns because those who express such views are citizens of liberal democratic regimes. Thus, populist anti-immigrant sentiments cannot simply be either dismissed or contained but should rather be taken seriously. One might object that liberal democratic legitimacy does not require taking all positions into account, including illiberal positions. However, the above-mentioned ambivalence of right-wing populism is crucial here. Indeed, populist right-wing parties can channel the claims of a moderate and more liberal electorate as well as blatantly illiberal and radical claims. Such illiberal anti-immigrant sentiments are nonetheless defended by parties that take part in representative legislative organs, including local administrations, national parliaments and the European Parliament, and have already been part of government coalitions (Akkerman, de Lange and Rooduijn 2016, pp. 1–5). As long as populist right-wing parties are admitted into electoral competitions, these underlying sentiments should not be ignored. It might be argued that, in a representative democracy, citizens express their preferences while voting, so taking into account such preferences through the electoral system is sufficient for democratic legitimacy. However, as Nadia Urbinati pointed out, representative democracy is better understood as a diarchy of will and opinion, where ‘the sovereign is not simply the authorised will contained in the civil law and implemented by states’ magistrates and institutions’ but is also ‘the opinion of those who obey and participate only indirectly in ruling’ (Urbinati 2014, p. 22). Thus, election day is not the only moment when citizens’ opinions matter: what citizens think and say outside the polling place should also be taken into account.

In the next section, I will argue that a realistic approach is needed to recognise the feasibility and legitimacy issues currently being raised by populist anti-immigrant sentiments and to determine what states and non-state actors should do to address them.

## Dealing with Anti-immigrant Sentiments in Normative Theorising: A Realistic Approach

According to Joseph Carens, normative theorists can situate their work along a continuum, from (extremely) realistic to (extremely) idealistic (Carens 1996, p. 169). Carens is mainly known for his arguments defending open borders, which are developed within an idealistic approach. Such an approach is useful for focusing on what justice requires in principle, under the best circumstances, because it allows one to transcend most practical constraints and to radically criticise the *status quo*. An idealistic approach permits us to see that the best realistic option may not correspond to what justice ultimately requires (Carens 1996, pp. 166–167). However, the more idealistic the approach is, the more current issues disappear from view (Carens 1996, p. 168). This is the case with populist anti-immigrant sentiments: since what justice in migration requires in principle does not depend on what citizens currently think or on which parties they vote for, an idealistic approach would then be unhelpful in acknowledging the feasibility and legitimacy issues raised by such illiberal sentiments.

A realistic approach, by contrast, takes institutional, political and social reality into account. It tries to identify the agents responsible for bringing about a change and to offer feasible proposals that have a plausible chance of being implemented under the current conditions. Gibney’s method aiming to ‘combine empirical and theoretical elements in an attempt to bring considerations of values and agency together’ (Gibney 2004, p. 16) can be considered an example of a realistic approach to the ethics of asylum. The first ten chapters of Carens (2013) also adopt a realistic approach. As the image of a continuum suggests, approaches can be more or less realistic and can include different factual conditions according to the goal and the scope of the inquiry.^15^

Note that a realistic approach to normative political theory is a methodological perspective, which need not be coupled with the endorsement of political realism^16^ and does not depend on the normative theory of justice that is assumed. A realistic approach is a standpoint allowing us to include relevant current factual conditions in liberal normative political theory. However, this does not mean that such a theory should be conservative rather than progressive or that it should accept social facts, such as popular preferences and opinions, as immutable (Lægaard 2006, p. 413). As Carens noted, a realistic approach is necessarily concerned with political feasibility. Realistic approaches discuss principles that can inform feasible political policies, consider how changes in migration policy might be brought about and identify agents capable of acting effectively (Carens 1996, pp. 159–160). Building on Carens’s discussion, I argue that a realistic approach allows political theorists to recognise populist anti-immigrant sentiments as feasibility constraints. However, moving beyond Carens’s view, I emphasise that a realistic approach does not necessarily assume anti-immigrant sentiments as given: it can, and should, offer strategies for liberal democracies to address that issue.^17^ Indeed, admitting the existence of feasibility constraints does not mean passively accepting them. Gilabert and Lawford-Smith propose a useful distinction between hard and soft feasibility constraints. Whilst hard constraints ‘rule out’ some options, soft constraints (e.g., economic, institutional and cultural ones) ‘place limits on what people are comparatively more likely to do, but the limits are neither permanent nor absolute’ (Gilabert and Lawford-Smith 2012, p. 813). Some existing feasibility constraints, they claim, not only can be but also ought to be loosened to achieve a morally desirable goal.

Concerning the ethics of migration, the morally desirable goal might be either a cosmopolitan goal (e.g., global equality of opportunity) or a goal that gives more weight to partialist claims (e.g., promoting social integration and peaceful interactions among citizens and immigrants who recognise each other as free and of equal moral worth). In both cases, I argue that widespread populist anti-immigrant sentiments represent a feasibility constraint. However, a realistic approach should include an empirically informed understanding of the kind of constraint at stake. I contend that illiberal anti-immigrant sentiments are a contingent rather than permanent feature of human societies, susceptible to amplification or reduction. Therefore, a realistic ethics of migration should examine how such sentiments can be effectively and legitimately addressed, instead of the sentiments simply being acknowledged and normative claims revised accordingly. If there is any realistic chance of persuading some citizens to temper or abandon their illiberal anti-immigrant sentiments, it is worth considering the ethics of the policies and practices that could bring about this change.^18^ As I will argue in the next section, face-to-face contact and context-sensitive narratives have a key psychological impact in both the formation and the transformation of anti-immigrant sentiments. Thus, local-level governments and non-state actors should be given more attention in a realistic ethics of migration that acknowledges anti-immigrant sentiments as a soft feasibility constraint.

Obviously, a realistic approach should idealise neither the malleability of public opinion nor the capacity of liberal-minded institutions and non-state actors to persuade the public. Empirical evidence, especially in social psychology, can offer clues as to what theorists can reasonably expect. Loosening the constraints posed by widespread illiberal anti-immigrant sentiments is likely to take time, and there may well be individuals who will not revise their anti-immigrant sentiments. Thus, tackling anti-immigrant sentiments may well be compatible with resistance policies and practices aimed at circumventing unjust immigration policies and practices in the short term.^19^ However, the aim of tackling anti-immigrant sentiments is to progressively achieve a change in public policy by curbing the spread of the illiberal anti-immigrant sentiments that lie behind the electoral success of populist right-wing parties and that result in policies and practices that are at odds with minimal liberal requirements.^20^

Taking a realistic approach is also methodologically fruitful for making sense of the legitimacy constraints that limit the admissible ways in which populist anti-immigrant sentiments may be approached. Indeed, a realistic approach is interested not in how a just receiving society for immigrants would look under ideal conditions but in assessing the legitimate ways in which change can be produced and which institutional or non-governmental actors should take action. Populist anti-immigrant sentiments cannot simply be neutralised, regardless of how this happens. A realistic ethics of migration should then discuss which policies would be compatible with the democratic legitimacy principles that require taking citizens’ views into account.^21^

In sum, taking populist anti-immigrant sentiments seriously requires a realistic approach to the ethics of migration that in turn allows theorists to see that such hostile attitudes represent feasibility constraints for any liberal ethics of migration. However, conceiving of populist anti-immigrant sentiments as soft constraints implies that they should not be taken as a given. Rather, such a conception will stimulate theorists to discuss how such hostile sentiments can be addressed. This shows the importance of assessing morally and politically legitimate ways in which populist anti-immigrant sentiments can be tackled, given that such sentiments express the views of a large share of citizens and are voiced by political parties that are admitted in democratic competitions, number among the representatives in parliaments and even participate in governments. The next section shows how a realistic approach that examines how anti-immigrant sentiments are formed and transformed also opens up a new level of theorising, namely, the ethics of local immigration policies, and brings to light the role of local governments and non-state actors.

## Shifting the Focus to Local Policies and Non-state Actors

Adopting a realistic approach can lead to a theoretically fruitful methodological change. By attracting theorists’ attention to how such hostile attitudes are formed and how they can legitimately be addressed, a realistic approach encourages theorists to engage with issues that have been underexplored. I will show two ways in which this methodological shift broadens the scope and breadth of normative theorising on migration.

First, this shift affects the level of theorising: once the issue of citizens’ sentiments towards immigrants comes to light, the importance of local-level policies in shaping the perceived impact of immigration emerges. Political theorists usually start with issues of justice in exclusion and inclusion within territorial and citizenship borders, thereby focusing on the admission and integration policies of central governments. By contrast, in adopting a realistic approach they will be encouraged to consider how local narratives and daily interactions affect migrants’ inclusion and the attitudes that citizens have towards them. Second, new actors will emerge alongside local governments: NGOs and other non-state actors either cooperate with local governments or independently carry out local initiatives that affect citizens’ sentiments towards immigrants. Although some authors have, for instance, considered immigrants as subjects with moral obligations (see Gibney 2019; Blake 2020), the main subject of the ethics of migration remains the state. Thus, bringing non-state actors into the picture is a promising result of adopting a realistic methodology. In what follows, I refer to some examples of local government policies and NGO initiatives addressing hostility towards immigrants. Far from proving that local policies and practices are always effective, these examples are meant to illustrate the theoretical potential of a realistic approach in considering how to address populist anti-immigrant sentiments instead of dismissing them or assuming them to be part of the *status quo*.

Before turning to non-state local actors, let us consider how a realistic approach highlights the importance of local-level policies. The role of local governments has largely been overlooked in regard to the ethics of migration and has not been explored in relation to how citizens form their sentiments towards immigrants.^22^ However, a realistic approach that takes widespread anti-immigrant sentiments into account would need to consider how citizens form such hostile attitudes and how immigrants’ presence impacts them. Normative theorists would thus be led to devote more attention to the local character of immigration, instead of debating admission and integration policies at the national level only. Indeed, social scientists and policymakers are increasingly aware of the fact that migrants’ social inclusion happens at the local level (Borkert and Caponio 2010, p. 9; OECD 2018, p. 24).

First, cities (and sometimes smaller towns and villages) are places where both natives and immigrants live (see de Shalit 2018, pp. 7–9; OECD 2018, pp. 24, 29). Thus, local governments have more detailed knowledge of their population and are better equipped to tailor inclusion policies. Second, the local level is the most appropriate scale to adopt when considering how citizens’ sentiments towards immigrants develop. In increasingly diverse areas, even citizens who do not have acquaintances who are immigrants necessarily have a sensory and emotional perception of the changing environment. Anti-immigrant sentiments can derive from a feeling of displacement due to ‘multicultural place-sharing’ (Wise 2010, p. 918): as the town landscape and population become increasingly unfamiliar, citizens may feel that ‘their sense of place is at risk’ (de Shalit 2018, p. 16). Furthermore, the local level is where direct contact (i.e., face-to-face interaction) between long-term residents and newcomers takes place.

Although interaction does not always discredit mutual prejudices and may possibly reinforce them,^23^ extensive research in social psychology demonstrates that positive intergroup contact generally reduces mutual hostility.^24^ It has been shown that interaction affects attitudes towards immigrants and preferences regarding immigration policy (McLaren 2003). Psychologists thus conclude that ‘promoting opportunities for positive contact, and hindering the potential for negative contact, both appear implementable strategies to improve relations between receiving society members and new immigrants’ (Kotzur et al. 2018, p. 824). Unsurprisingly, several local governments rely on contact theory to reduce hostility towards immigrants and improve integration.

In the United States, for instance, over a hundred local governments have joined the Welcoming America network. Welcoming America claims that the focus of integration policies should not be limited to immigrants only: governments and non-state actors are encouraged to give more attention and resources to immigrant-receiving communities (Jones-Correa 2011). In Europe, too, municipalities are increasingly aware of the importance of promoting integration at the local level and have begun to devise policies directed at citizens, although immigrants still seem to be the main target of such efforts. In the UK, for instance, the Inclusive Cities network helps five cities and their local partners ‘achieve a step-change in their approach towards the integration of newcomers locally’ (Broadhead 2017) and has launched a learning exchange with two US cities that are part of the Welcoming America network (Broadhead 2018b). By contrast, national immigration and integration policies still largely fail to address anti-immigrant sentiments and to adopt specific policies to promote positive interaction. Thus, these kinds of policy goals and choices may remain unnoticed by political theorists if they only consider national-level policies.

Local governments currently seem more active than national governments in addressing anti-immigrant sentiments. Moreover, there may be good reasons to focus on the local scale when moving to the normative issue of what a liberal state should do (or might be legitimately permitted to do) to tackle populist anti-immigrant sentiments. Clearly, illiberal anti-immigrant sentiments are also widespread at the local level (despite the intensity and capillarity of those sentiments varying from one local community to another). In addition, local governments are not necessarily hospitable to migrants or eager to counter illiberal anti-immigrant sentiments: right-wing populist parties may well be in power in local governments too. The point is that the local context provides a more favourable environment for those local governments that are not led by right-wing populists to counter illiberal anti-immigrant sentiments in their area. Indeed, local-level policies are better suited than national-level policies to address both the feasibility and legitimacy challenges that populist anti-immigrant sentiments pose to the implementation of any immigration and integration policy consistent with the basic liberal values of freedom and equality. To put it simply, policies aimed at persuading citizens to adopt more liberal attitudes towards migrants have a better chance of being effective and legitimate when carried out locally.

First, as feasibility (soft) constraints, such illiberal hostile sentiments may be more effectively counteracted through local-level policies, such as those inspired by contact theory, that foster positive direct interactions between citizens and immigrants. Furthermore, even communication strategies aimed at dispelling anti-immigrant rumours and populist anti-immigrant rhetoric may be more effective if carried out locally.^25^ Research shows that citizens tend to overestimate the size of the immigrant presence, as well as the economic, social and cultural costs that such presence may entail for the receiving state.^26^ Though not necessarily related to the actual number of immigrants living in an area (Rustenbach 2010, p. 65), anti-immigrant sentiments seem related to the perception of a large immigrant presence.^27^ However, merely providing information may not result in the debunking of stereotypes and myths (Hidalgo 2019a, p. 189). Abstract statistics and quantitative data on migration may be too distant from citizens’ situated experience (Epstein et al. 2014). Narratives, by contrast, may be more effective, particularly if carried out at the local level and including references to the specific context and residents’ lived experiences (Broadhead 2018a). Indeed, storytelling allows vicarious contact, a form of indirect interaction that has been proven to reduce intergroup anxiety and prejudice even in segregated conflictual areas, thus facilitating future direct interaction (Vezzali et al. 2014). As I. M. Young suggested, narratives need not substitute arguments, but they can be combined with arguments or offer a starting point when shared premises are missing (Young 2000, p. 72). In sum, there is good reason to think that persuading citizens to revise their anti-immigrant sentiments is more effective when policies are sensitive to the local context. Furthermore, even when right-wing populist parties are in power in the central government (or exert a strong influence over it), liberal and progressive parties can be elected in local governments and can still promote inter-group interaction and devise context-sensitive narratives to counter populist right-wing rhetoric.

Second, the role of local governments emerges as particularly important in regard to the democratic legitimacy issues that anti-immigrant sentiments raise. As I have argued in ‘Populist Anti-immigrant Sentiments in the Ethics of Migration’ section, the widespread anti-immigrant sentiments promoted by populist right-wing parties should not be merely contained. They need to be taken seriously by policymakers, as well as by political theorists who are interested in the normative implications regarding the accessibility of any liberal theory of just immigration policies. Taking anti-immigrant sentiments seriously means that those who hold such hostile sentiments should not simply be called ignorant and racist and thus be excluded but should rather be approached with a dialogical attitude so that potential legitimate underlying issues and claims can emerge, be heard and disentangled from the scapegoating of migrants.

For instance, hostility towards immigrants may be connected to forms of misrecognition or marginalisation: some citizens feel that they have been forgotten by political institutions and that their voices are no longer heard or no longer matter. They may also blame immigrants for phenomena such as unemployment, housing shortages or urban blight, which actually have deeper and more complex causes than the (actual or perceived) large presence of immigrants. Furthermore, economic anxiety is strictly intertwined with existential cultural anxiety. Some people may feel not only economically but also culturally left behind as their traditional values become increasingly marginalised in public culture. Inglehart and Norris (2016) warn that the rise of populism should not be attributed to economic inequality alone: populist messages may be particularly appealing for those individuals who resent being told that their values are ‘politically incorrect’ and feel they have become strangers from the dominant culture in their own country. Importantly, both material and cultural concerns may relate to members of an individual’s in-groups (e.g., neighbours, colleagues, relatives, fellow citizens), including future generations.^28^ Thus, those who hold illiberal anti-immigrant sentiments and are susceptible to populist right-wing rhetoric do not necessarily belong to marginalised or economically destitute social groups.

If tackling anti-immigrant sentiments in a legitimate and effective way requires a dialogical attitude, neighbourhoods and towns seem to be appropriate venues through which institutions can act. Local administrations may provide fora where citizens can express their views and concerns about immigration and feel that their perspective is being seriously taken into account by their interlocutors. In fact, given the smaller scale of their constituent population, local governments are well situated to engage in a bidirectional dialogue with their citizens and to publicly discuss immigration and integration policies and their impact on the local community. Even when local governments have to comply with national laws or accept decisions made elsewhere, they can still play a significant role in helping their citizens cope with change and reducing conflict. For instance, in Lewiston, Maine, residents initially felt that their little town would be overwhelmed by the sudden arrival of resettled Somali refugees in 2001. The municipality, however, engaged in ‘dispelling myths about them and explaining the circumstances that prompted their move to the United States’. The efforts of the local government addressed anxiety and resulted in more welcoming attitudes (Jones-Correa 2011, pp. 6–7, 24–25).

I have argued so far that a realistic approach encourages theorists to include local governments when discussing how and why policymakers should take into account the claims of those who hold illiberal anti-immigrant sentiments and what kind of policies they might legitimately enact to persuade citizens to adopt more liberal attitudes towards newcomers and immigrant minorities. However, local governments are not the only actors involved: once theorists adopt a realistic approach and shift the focus from national-level policies to the local scale, non-state actors also emerge. Although non-state actors cannot enact policies themselves, they can still have a relevant impact on public opinion. NGOs may successfully cooperate with liberal-minded local governments in providing both policy advice and practical expertise for organising a wide range of activities, as Welcoming America’s long-term experience shows. Members of Welcoming America have promoted town hall meetings in different locations (e.g., in Littleton, Colorado, and in the Philadelphia region), and these meetings have gathered a wide range of participants, including immigrants and citizens who favour restrictions on immigration. Other programmes have involved one-on-one mentorship for newcomers that is aimed at creating lasting inter-group relationships, in addition to improving immigrants’ English skills (see Jones-Correa 2011).

One might object that not every local government would be willing to cooperate with NGOs to address the illiberal anti-immigrant sentiments that lie behind the success of several right-wing populist parties. At the very least, local administrations run by populist right-wing parties are unlikely to endorse communication campaigns aimed at countering their own narratives. However, the lower the level of engagement of local governments is, the more visible the impact of civil society becomes. Civil society organisations can both engage in communication strategies and promote positive personal interactions through local-level initiatives, even when local institutions are far from welcoming to immigrants. A research project co-funded by the EU mapped over 300 community-building initiatives run by civil society organisations across nine European countries and identified the ‘best practices’ for preventing racism and xenophobia, particularly against forced migrants, through ‘encounters between people and social inclusion’ (Doyle 2017, p. 5). Civil society organisations might promote justice in migration in alternative ways, for instance, by assisting migrants and resisting unjust immigration policies. Again, addressing citizens’ hostility towards migrants is not incompatible with such actions: the same NGO may even engage in both endeavours. However, more theoretical attention should be devoted to the ethics of civil society involvement in tackling illiberal anti-immigrant sentiments and to the cooperation between NGOs and institutions.

This section has illustrated two ways in which a realistic methodological standpoint broadens the scope of normative theorising regarding the ethics of migration. In exploring how anti-immigrant sentiments are formed and how they can be addressed, such an approach shifts the focus from the state to the local level. This is an important methodological upshot, given that the ethics of immigration are usually concerned with the policies of central governments. Moreover, such a realistic and local approach brings to light other actors in addition to local governments: non-state actors such as NGOs also take action, either in cooperation with state actors or autonomously, in ways that influence citizens’ sentiments. Normative political theorists are therefore urged to consider what role such non-state actors should play in addressing populist anti-immigrant sentiments.

## Conclusion

This paper has argued that theorists working on the ethics of migration should take the rise of populist right-wing movements and the diffusion of anti-immigrant sentiments in Western liberal democracies seriously. First, widespread hostile attitudes towards immigrants impose serious feasibility constraints on the implementation of any liberal immigration and integration policy, not only the most cosmopolitan ones. Moreover, democratic principles require taking the opinion of right-wing populist parties’ electorate into account. Thus, populist anti-immigrant sentiments should be neither dismissed nor simply contained.

I have argued that a realistic approach to the ethics of migration allows political theorists to include populist anti-immigrant sentiments within normative theorising and to acknowledge that there are important feasibility and legitimacy reasons why such sentiments cannot be ignored. Nevertheless, I also contended that widespread anti-immigrant sentiments (which are both fuelled and exploited by populist right-wing parties) are a contingent feature of contemporary liberal democratic societies. Thus, a realistic approach should not take them as a given and then revise the relevant principles accordingly. To the contrary, such an approach allows theorists to discuss how to address the challenge that populist anti-immigrant sentiments pose to the implementation of any liberal theory of justice in migration. Finally, I have argued that adopting a realistic approach and bringing populist anti-immigrant sentiments into the picture offers the opportunity to extend the scope of normative theorising on the ethics of immigration and integration policies. I have illustrated how being realistic about the psychological and social processes behind the formation and transformation of anti-immigrant sentiments can lead political theorists to discuss the role of local governments and non-governmental actors in addressing their citizens’ hostility towards immigrants.

## Footnotes

1 To cite a few recent examples, in 2017, although finally defeated, the National Front candidate, Ms. Le Pen, participated in the run-off during the French presidential elections, while the Dutch Party for Freedom increased its seats in Parliament, and in Austria over a quarter of the electorate voted for the Freedom Party (FPÖ), which then entered the government. In 2018, during Italian elections, the right-wing populist League Party proved to be the third most prominent political party (the first party being the populist Five Star Movement), and its leader was appointed deputy-prime minister and home affairs minister; the following year, the League Party became the most voted for Italian party in the MEP elections.

2 For instance, in 2018, Mr. Orban’s party, Fidesz, triumphed in Hungary for the third time, while almost a fifth of the electorate voted for the even more radical Movement for a Better Hungary (Jobbik).

3 According to Müller (2016), populism is also anti-pluralist since populists claim to exclusively represent the general will of a homogeneous people.

4 Several scholars have attempted to identify the features marking the ‘common denominator’ of populism, although there is no consensus about a basic definition (for a recent critical review, see Barker 2020). For history of the concept, see Skenderovic 2017.

5 Left-wing Latin American populism also stresses a common identity as natives of Spanish America in opposition to the foreign imperial power (this applies to both ‘classic’ and contemporary ‘third-wave’ ‘radical’ populism: see Rovira Kaltwasser 2014; de la Torre 2017). However, Latin American populism, even when appealing to ethnicity and indigenous identity, adopts an inclusive conception of the people, while European right-wing populism is typically exclusive (see Madrid 2008; Mudde and Rovira Kaltwasser 2013, p. 167).

6 It has recently been argued that the normative claims and empirical assumptions defended by Miller and other liberal nationalists are not significantly different from the claims of right-wing populists, thus turning out to also be illiberal and biased by racism (Finlayson 2020). My aim here is neither to discuss how much nationalism is admissible for liberal nationalism to remain coherent with its liberal assumptions, nor to set precise reasonableness limits on the ethics of immigration. My point is rather to emphasise that widespread populist anti-immigrant sentiments are clearly illiberal and morally problematic even for liberal nationalists.

7 Donald Trump famously defended a ‘zero-tolerance’ policy against incoming undocumented migrants and strongly opposed his predecessor’s policies on granting citizenship to ‘Dreamers’ (long-term undocumented residents who settled in the US as children). Populist right-wing leaders in Europe have also claimed that unauthorised migrants (including potential asylum seekers) should be indiscriminately expelled and, ideally, kept out of their nation’s borders. When in power, they have built electrified fences, impeded rescue operations at sea and forbidden rescued migrants from disembarking (see Dunai 2017; Pianigiani et al. 2018).

8 On Trump’s statements, see Korte and Gomez (2018).

9 Unsurprisingly, populist right-wing leaders have recently been blaming immigrants for spreading COVID-19: see Trilling 2020.

10 A well-known example is the ‘Muslim travel ban’ introduced by Trump in the US; however, similar restrictions were invoked by European populist leaders, such as the Dutch Geert Wilders (Tait 2017).

11 Populist right-wing rhetoric often instigates Islamophobia and invokes restrictions on Muslims’ freedom of worship. For instance, Geert Wilders in the Netherlands and Matteo Salvini in Italy claimed that Islam is incompatible with public culture. Wilders also pushed for the closure of mosques and the banning of the Quran (Mortimer 2016).

12 Miller (2016a, p. 131). However, a brief survey of how the hostility towards migrants prompted the success of populist parties and leaders is offered in Parvin’s comment to Miller’s book (Parvin 2017). A few references to the rise of right-wing populism and to its xenophobic rhetoric can also be found in Song (2019, pp. 5, 167).

13 Gibney (2004, pp. 17, 22) includes two explicit references to the rise of ‘far-right leaders’ and particularly to the growing electoral success of the National Front in France. However, addressing the spread of hostile attitudes towards migrants and asylum seekers is not the aim of the book.

14 Parvin (2017) notes that ‘justice for Miller needs to be realistic in the sense that it needs to accord with overarching, widespread ideas shared among the people to whom it will apply’. Indeed, Miller (2016a) quotes opinion polls reporting that many citizens in various countries favour immigration restrictions. In Miller’s view, this fact supports his claim that a strong cosmopolitanism would ignore partiality in moral obligations. However, note that his account of the obligations to refugees grounded on human rights, as well as the obligation to provide reasons for refusing admission to migrants whose human rights are not at risk, are incompatible with widespread illiberal anti-immigrant sentiments.

15 Sandelind (2019, p. 335) observes that the continuum metaphor obscures how normative theories adopting an idealistic approach ‘often turn out not to be *less* concerned with facts, but concerned with *different* facts’. Fine (2017) also remarks that Miller’s realistic approach leaves out important real-world facts, particularly past injustices that lie behind existing territorial borders and population distributions. Indeed, both the quantity and the quality of the presupposed facts matter: a theory meant to be realistic can turn out to be idealised if it fails to incorporate relevant facts or if it makes idealised assumptions, mixing up ideal and non-ideal components (see Lægaard 2016); conversely, a good idealistic theory is not necessarily idealised if it includes the real-world presuppositions pertinent to that degree of abstraction.

16 Political realism is not merely a methodological approach but rather a philosophical tradition having a distinct conception of politics and political theory (Rossi and Sleat 2014; Sleat 2016). In the recent literature, some authors have used two different meanings of realism in political theory. According to David Miller, realism_1_ is methodological and contrasted with utopianism, while realism_2_ is a substantive position contrasted with political moralism (Miller 2016c, pp. 218–220). I suggest that what Miller calls realism_1_ (i.e., a methodological realism) would be better described, in Carens’s terms, as a ‘realistic approach’, while the term ‘realism’ would apply to political realism only.

17 This is set aside in both Carens (1996) (see p. 160) and Carens (2013).

18 Hidalgo (2019a, p. 188) also argues that ‘it’s possible to change minds on immigration’. In addition, successful populist anti-immigrant rhetoric also depends on the malleability of sentiments.

19 Individuals, either alone or in civil society organisations, can disobey or subvert the implementation of particular immigration policies. There is a growing body of literature on the ethics of resistance by citizens and by immigrants themselves; see Blunt (2019); Hidalgo (2019a, b). Local governments may also exert some autonomy in their jurisdiction. Sanctuary cities, for instance, challenge the enforcement of the immigration policies issued by the central government, e.g., by refusing to cooperate in deportations. On the ethics of sanctuary policies, see Blake and Hereth 2020; Lenard 2019.

20 Some cases of resistance might also count as civil disobedience, and thus potentially contribute to changing public opinion and institutional policies. However, not all acts of resistance count as civil disobedience, nor are all forms of civil disobedience equally promising in tackling anti-immigrant sentiments and avoiding populist backlash (see Hidalgo 2019b).

21 I have examined this issue in Santi Amantini (2020﻿).

22 An exception is the ‘urban’ approach to the ethics of migration adopted by de Shalit (2018). It is worth noting that sanctuary cities have begun to attract increasing attention among political philosophers. However, as I argued in footnote 19, sanctuary policies are examples of local-level policies aiming to resist the implementation of unjust immigration policies, rather than to tackle anti-immigrant sentiments.

23 Negative contact strongly reinforces hostility because out-group membership (e.g., being an immigrant) is perceived to be salient in negative interactions, while similarities tend to be stressed during positive interactions. However, previous positive interactions can buffer against future negative ones (Paolini et al. 2014).

24 Intergroup contact theory, first formulated by Williams in 1947, was developed in Allport’s seminal work, *The Nature of Prejudice* (1954). An extensive meta-analysis based on 713 independent samples from 515 studies confirms that intergroup contact typically reduces intergroup prejudice, even when highly conflictual and unequal background conditions apply (Pettigrew and Tropp 2006).

25 Information helps shape citizens’ attitudes towards immigrants, and it has a great impact when direct contact with immigrants is rare (Vezzali et al. 2014, p. 346). Indeed, when direct contact experiences are scarce or unavailable, people have to rely on indirect experiences as a source of information for attitude formation (Vezzali et al. 2014, p. 368; on attitudes towards immigrants, see Herda 2010, p. 688).

26 A decade ago, in a survey carried out in 21 European countries, participants estimated the size of the immigrant population to be, on average, double its actual size (Herda 2010, Table 2). A recent study based on Eurostat data confirmed that, on average, European citizens estimated immigrants to be nearly 17% of the population, while they were actually 7.2%. In the most extreme case (i.e., Italy) the perceived size of the immigrant presence was more than 17 points higher than the real percentage (Valbruzzi, 2018).

27 Innumeracy rates and anti-immigrant sentiments proved strong even among people who live in low-immigration areas. Moreover, by contrasting this data with the NIM index elaborated by the Pew Research Centre to measure the nationalist, anti-immigrant and anti-religious minority sentiments, a correlation has been found between the innumeracy rate and the level of hostility towards immigrants (Valbruzzi 2018). In a Belgian survey, too, anti-immigrant sentiments were positively correlated with the perception of a high number of immigrants, while living in a high-immigration area proved to have no significant correlation with anti-immigrant sentiments (Hooghe and de Vroome 2015).

28 For a discussion of both economic- and identity-related concerns associated with populism, see Mounk (2018). On how people’s sense of personal experience may encompass the experiences of another member of the group, see also Polletta and Callahan (2017).
